# Postoperative Solitary Brain Metastasis from Residual Gastric Cancer: A Rare Case Report

**DOI:** 10.70352/scrj.cr.25-0306

**Published:** 2025-09-02

**Authors:** Masaya Matsumoto, Kojiro Eto, Satoshi Ida, Hiroki Tsubakihara, Keisuke Kosumi, Kazuto Harada, Yuji Miyamoto, Ken Uekawa, Akitake Musaka, Masaaki Iwatsuki

**Affiliations:** 1 Department of Gastroenterological Surgery, Graduate School of Medical Sciences, Kumamoto University, Kumamoto, Kumamoto, Japan; 2 Department of Neurosurgery, Graduate School of Medical Sciences, Kumamoto University, Kumamoto, Kumamoto, Japan

**Keywords:** residual gastric cancer, solitary brain metastasis

## Abstract

**INTRODUCTION:**

Brain metastasis from gastric cancer is rare (0.5%) and often occurs with metastasis to other organs. We herein describe a very rare patient with a solitary brain metastasis from residual gastric cancer with no metastasis to other organs.

**CASE PRESENTATION:**

The patient was an 82-year-old man who visited a previous institution for anemia. Upper gastrointestinal endoscopy revealed a type 2 tumor extending from the esophagogastric junction to the upper part of the residual gastric body. Biopsy revealed tubular differentiated adenocarcinoma, and he was referred to our institution. He had a history of distal gastrectomy for a gastric ulcer in his 30s. After contrast-enhanced CT, we diagnosed residual gastric cancer (cT4aN + M0 cStage III). After three courses of preoperative chemotherapy with S-1 plus oxaliplatin, the patient underwent open total resection of the residual stomach, lower esophagectomy, D2 dissection, and Roux-en-Y reconstruction and was discharged without postoperative complications. Six months after surgery, thoracic and abdominal contrast-enhanced CT showed no apparent recurrence. However, 1 month later, he began to experience speech difficulties and mobility issues, and head CT revealed a 3-cm tumor in the left frontal lobe. After whole-body contrast-enhanced CT and PET-CT, the brain tumor was confirmed as a solitary lesion with no metastasis to other organs. The patient underwent open brain tumor resection, and pathology diagnosed brain metastasis from residual gastric cancer. Postoperatively, he underwent radiation therapy (40 Gy in 8 fractions) to the tumor cavity. At the time of writing, 24 months have passed since the gastrectomy and 16 months have passed since the removal of the brain tumor, with no significant neurological damage or other evidence of distant metastasis.

**CONCLUSIONS:**

We experienced an extremely rare case of a solitary brain metastasis after residual gastric cancer surgery. Our findings suggest that aggressive local treatments for brain metastasis, including surgical resection and radiotherapy, may contribute to improvements in symptoms and prognosis.

## Abbreviations

SUVmax
: maximum standardized uptake value
STI
: stereotactic irradiation
WBRT
: whole brain radiation therapy

## INTRODUCTION

Although the frequency of metastatic brain tumors has recently increased,^1)^ brain metastases from gastric cancer are relatively rare, accounting for only 0.47%–0.7% of cases.^2)^ Brain metastases from gastric cancer are often accompanied by metastases to other organs at the time of diagnosis; however, brain metastasis as a single entity is rare.^3–5)^

Residual gastric cancer can lead to unexpected recurrence and metastasis owing to changes in lymphatic and blood flow resulting from surgical invasion.^6)^

We report, to the best of our knowledge, the first case of a solitary brain metastasis from residual gastric cancer, without metastasis to other organs, with a review of the literature.

## CASE PRESENTATION

**Fig. 1:**
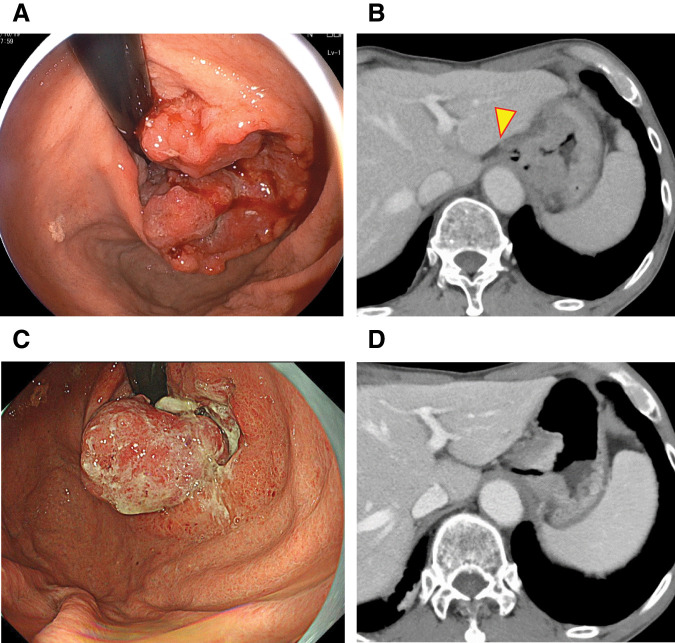
Imaging findings before and after preoperative chemotherapy (**A**) Upper gastrointestinal endoscopy before preoperative chemotherapy showing a type 2 tumor at the esophagogastric junction. (**B**) Contrast-enhanced CT before preoperative chemotherapy showing wall thickening mainly at the esophagogastric junction and (the yellow arrowhead) local partial invasion of the left diaphragmatic crus. (**C**) Upper gastrointestinal endoscopy after preoperative chemotherapy showing a slight reduction in tumor size. (**D**) Contrast-enhanced CT after preoperative chemotherapy showing slight improvement in the tumor size and wall thickening, with no distant metastases or new lesions.

The patient was an 82-year-old man with a history of pyloric gastrectomy with Billroth-I reconstruction for a gastric ulcer in his 30s. Upper gastrointestinal endoscopy was performed to investigate anemia, and a type 2 tumor was found extending from the esophagogastric junction to the upper part of the residual gastric body (**Fig. 1A**). Contrast-enhanced CT showed wall thickening in the same area and local invasion of the left diaphragmatic crus was suspected (**Fig. 1B**). Therefore, surgery was planned after three courses of preoperative chemotherapy with a combination of S-1 plus oxaliplatin, which comprised a 3-week course of S-1 (100 mg/day) orally on days 1–14, with oxaliplatin (130 mg/m^2^) intravenously on day 1.^7)^ Upper gastrointestinal endoscopy and contrast-enhanced CT of the thorax and abdomen before and after preoperative chemotherapy showed that the tumor at the esophagogastric junction had decreased slightly in size, with no distant metastases or new lesions (**Fig. 1C**, **1D**).

**Fig. 2:**
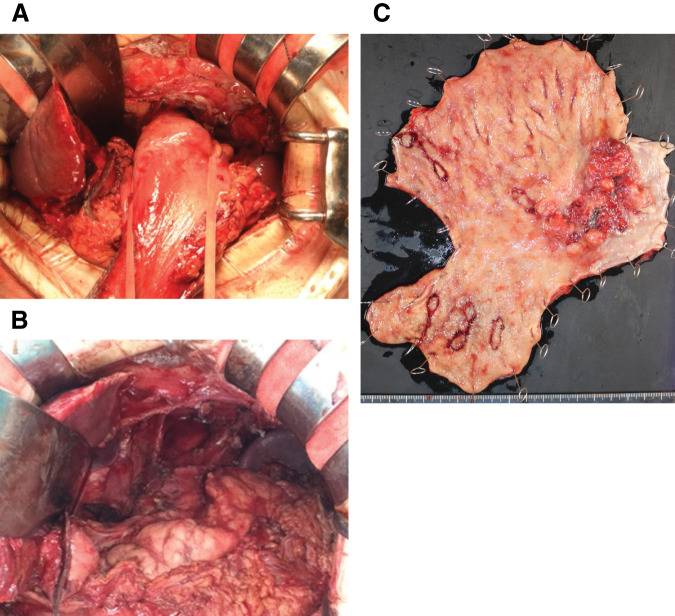
Intraoperative findings and postoperative specimens for primary tumor (**A**, **B**) Intraoperative photographs before and after total residual gastrectomy. (**C**) Gross specimen. The diagnosis was GE, type2, B-47-O distal gastrectomy B-I, tub1, ypT3(SS), INFb, Ly1a, V1b, pPM0(10 mm), pDM0(18 mm), CY0, pN0. ypT3N0M0 ypStage IIA.

After three courses of chemotherapy, open total resection of the residual stomach, lower esophagectomy, D2 dissection, and Roux-en-Y reconstruction were performed (**Fig. 2A**, **2B**). There were no complications, and the patient was discharged on POD 13. Postoperative pathologic examination revealed GE, type2, B-47-O distal gastrectomy B-I, tub1, ypT3(SS), INFb, Ly1a, V1b, pPM0(10 mm), pDM0(18 mm), CY0, pN0, ypT3N0M0 ypStage IIA (**Fig. 2C**). After discussing the results with the patient and his family, the decision was made not to provide postoperative adjuvant therapy, considering postoperative deterioration of general condition and his advanced age.

**Fig. 3:**
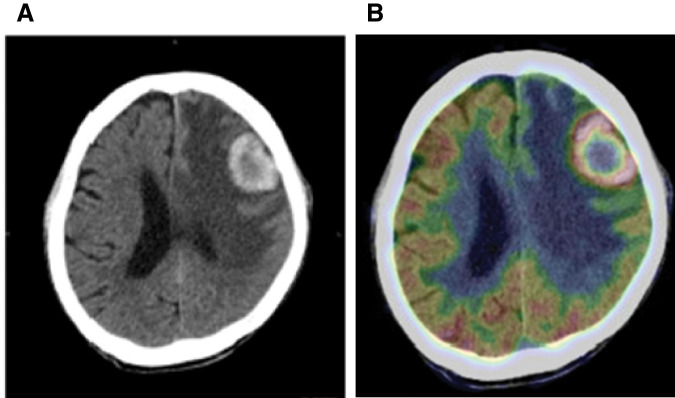
Imaging findings at the time the brain tumor was identified (**A**) Plain CT showing a neoplastic lesion in the left frontal lobe with hemorrhage and surrounding edema. (**B**) PET-CT showing an abnormal accumulation, with an SUVmax = of 10.1 around the margins of the mass. No other distant metastases or new lesions were noted.

**Fig. 4:**
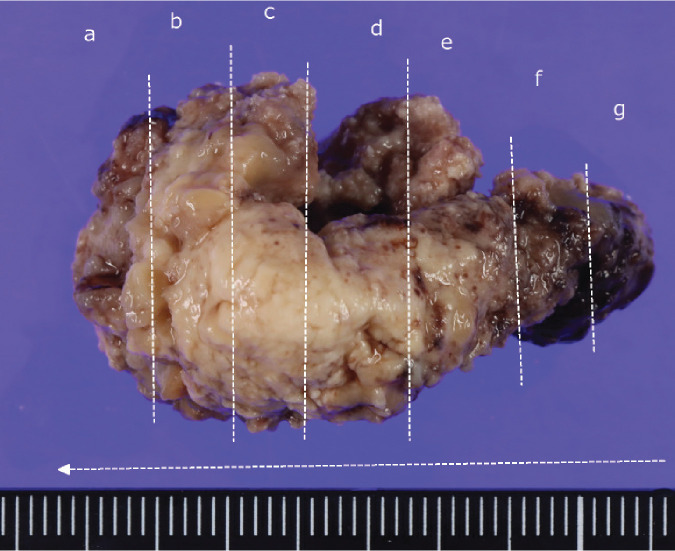
Postoperative specimens for brain tumor.

Contrast-enhanced CT of the thorax and abdomen 6 months postoperatively showed no apparent recurrence or distant metastasis. However, 1 month later, he began to experience speech difficulties and mobility issues. Head CT revealed a 3-cm tumor in the left frontal lobe, and PET-CT showed remarkable abnormal accumulation, with a maximum standardized uptake value of 10.1 (**Fig. 3**) only at the brain site. These examinations revealed no other distant metastases or new lesions. Open brain tumor resection was performed for the solitary brain tumor, with postoperative tumor cavity irradiation (40 Gy in 8 fractions) (**Fig. 4**). After treatment, his speech difficulties and mobility issues improved, and he was able to walk on his own.

**Fig. 5:**
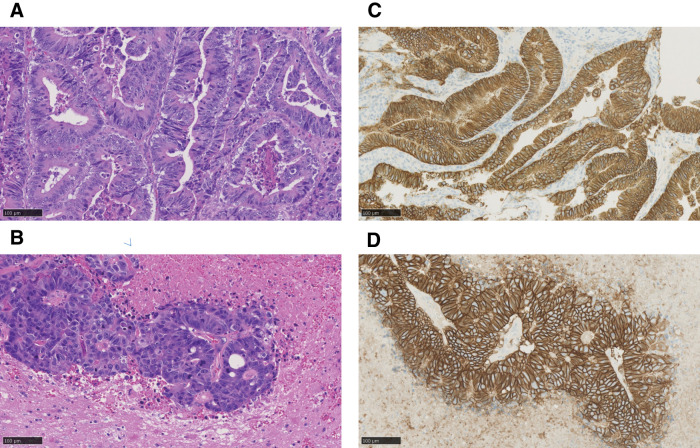
Histopathological image of the excised primary and brain tumor (**A**) Cuboidal or hypercylindrical atypical cells are seen proliferating with irregular glandular duct structures. (**B**) Consistent with the pathology of residual gastric carcinoma, a solitary brain metastasis of residual gastric carcinoma was suspected. Stain: hematoxylin and eosin; Magnification: ×200. (**C**, **D**) Both the primary tumor and brain metastasis were HER2-positive, suggesting a high probability that they originated from the same tumor.

Postoperative pathology findings were similar to those for the residual gastric carcinoma (**Fig. 5A**, **5B**). Both the primary tumor and brain metastasis were HER2-positive, suggesting a high probability that they originated from the same tumor (**Fig. 5C**, **5D**). On the basis of the pathological findings and the patient’s clinical course, the brain tumor was diagnosed as brain metastasis from residual gastric cancer. After consulting with the patient and his family, postoperative chemotherapy was not administered. At the time of writing, 16 months has passed since the brain tumor resection with no apparent recurrence or neurological symptoms.

## DISCUSSION

We described a case of solitary brain metastasis 7 months after residual gastrectomy, in which the patient underwent open brain tumor resection and postoperative tumor cavity irradiation and survived recurrence-free for at least 16 months. We performed PubMed search from 2000 to 2025, using terms such as “brain metastasis,” “residual gastric cancer,” and “without metastasis to other organs,” but no reports were found. To the best of our knowledge, this is the first report of a case of a solitary brain metastasis from residual gastric cancer without metastasis to other organs.

Previous reports indicate that brain metastases from gastric cancer may develop within one year postoperatively; however, cases with a latency of 2–4 years have also been reported.^4–8)^ In early gastric cancer, brain metastases have been observed after a longer period, reporting variability in the timing of onset.^5)^ The timing of brain metastasis from residual gastric cancer remains controversial.

Harada et al. reported that brain metastases occur in only 2.9% of cases involving upper gastrointestinal cancers, highlighting their rarity. A detailed analysis of the authors’ findings revealed that all such cases were esophageal cancer or esophagogastric junction (EGJ) cancer; no case of gastric cancer or residual gastric cancer was observed. Most cancers of the upper gastrointestinal tract that metastasize to the brain are esophageal cancers or EGJ cancers, although the precise mechanism remains unclear.^9)^ We performed PubMed search from 2000 to 2025, using terms such as “brain metastasis” and “esophagogastric junctional cancer.” We identified 12 cases of brain metastasis from EGJ cancer, including our case. Excluding our case, all cases showed other organ metastasis or lymph node metastasis. Brain metastases are thought to occur through three primary routes: hematogenous spread via the portal venous system, retrograde hematogenous spread through the vertebral venous plexus, and lymphatic spread from lymph nodes near the primary tumor. Brain metastases are often accompanied by metastases to the liver or lungs.^3)^ However, in our case, no metastases to lymph nodes or other organs were observed, suggesting retrograde hematogenous spread via the vertebral venous plexus. Accordingly, the metastatic pathway in the present case was possibly from the esophageal vein to the vertebral venous system via the azygos vein because the primary lesion was EGJ cancer.^10)^ It is also possible that alterations in lymphatic or blood flow caused by surgical intervention for residual gastric cancer contribute to an atypical metastatic pathway.^5–7)^

The 2024 Japanese metastatic brain tumor treatment guidelines recommend tumor resection, stereotactic irradiation (STI), whole brain radiation therapy (WBRT), or a combination of both.^11)^ The guidelines recommend resection plus STI for cases with a single or a few (2–4) brain metastases where the patient is in good general condition and resection is expected to improve function. For cases with multiple (5 or more) metastases, WBRT is generally recommended, but resection or STI may also be added in cases where functional improvement or improved prognosis is expected. WBRT is associated with concerns about late adverse events, such as encephalopathy and cognitive dysfunction, and STI is becoming more common owing to recent improvements in radiotherapy techniques. Mahajan et al. reported a significantly higher rate of local control in their STI group compared with the follow-up group after resection of metastatic brain tumors (STI: 72%, Follow-up: 43%).^12)^ In a meta-analysis of gastric cancer brain metastases, only about 10% of cases underwent surgery for brain metastases, but in cases where resection was performed, there was a suggestion of improved prognosis compared with non-resection cases. Considering the guidelines, in gastric cancer brain metastases, resection should be actively considered for solitary or few lesions if the general condition permits, and STI should be added if possible.^13)^ In our case, the brain metastasis was solitary, with no other metastases. With these considerations, we performed STI (40 Gy/8fr) to the tumor cavity after tumor resection in our case.

In general, the local effects of chemotherapy for metastatic brain tumors are inferior to those of radiation therapy or tumor resection. There are case reports demonstrating the efficacy of oral fluoropyrimidine drugs.^14)^ Trastuzumab has poor brain penetration due to the blood–brain barrier. Monoclonal antibody drugs, which are high-molecular-weight compounds, do not cross the blood–-brain barrier and therefore typically do not exhibit efficacy against metastatic brain tumors when administered systemically. Immune checkpoint inhibitors have been reported to exhibit synergistic effects with radiation therapy in gastric cancer brain metastases.^15)^

A comprehensive analysis by Harada et al.^9)^ revealed that the overall survival of patients with brain metastases from gastric cancer is approximately 18 months, reflecting a generally poor prognosis. However, the study also suggested that patients with a solitary brain metastasis or without concomitant metastases to other organs tend to have relatively better outcomes. Furthermore, patients who underwent surgical resection for brain metastases demonstrated a potential improvement in prognosis.^5)^ In the present case, the patient has been recurrence-free and without neurological symptoms for 16 months following brain tumor resection, at the time of writing. Although the status of metastasis and the patient’s general condition must be taken into account, aggressive treatment for brain metastasis, if possible, may help maintain QOL and improve the prognosis.

## CONCLUSIONS

We experienced an extremely rare case of a solitary brain metastasis after residual gastric cancer surgery. Our findings suggest that aggressive local treatments for brain metastasis, including surgical resection and radiotherapy, may contribute to improvements in symptoms and prognosis.

## DECLARATIONS

### Availability of data and material

All data generated or analyzed during this study are included in the published article.
